# Use of modern contraceptive methods and its association with QOL among Nepalese female migrants living in Japan

**DOI:** 10.1371/journal.pone.0197243

**Published:** 2018-05-16

**Authors:** Richa Shah, Junko Kiriya, Akira Shibanuma, Masamine Jimba

**Affiliations:** Department of Community and Global Health, Graduate School of Medicine, The University of Tokyo, Tokyo, Japan; University of West London, UNITED KINGDOM

## Abstract

**Introduction:**

Modern contraceptive use among migrants may be limited due to factors such as language barriers, socio-economic condition, and inadequate knowledge. Limited evidence is available on the factors associated with the use of modern contraceptive methods by migrants in Japan. Moreover, evidence remains scarce on the quality of life (QOL) of migrants in relation to contraceptive use. As the Nepalese migrant population in Japan is increasing, this study was conducted to address their sexual and reproductive health issues. Therefore, the objectives of this study were to examine the factors associated with the use of modern contraceptive methods and to explore the association between the use of modern contraceptive methods and QOL among married Nepalese female migrants in Japan.

**Methods:**

This is a cross-sectional study conducted among 189 married Nepalese female migrants residing in five prefectures of Japan from August to October 2016. This study employed snowball sampling to recruit the migrants. Simple and multiple logistic analyses were used to examine the association between independent variables and the use of modern contraceptive methods. Similarly, multiple linear regression analysis was used to examine the association between the use of modern contraceptive methods and quality of life.

**Results:**

Migrants who had higher knowledge of modern contraceptive methods were more likely to use them (Adjusted Odds Ratio [AOR] = 1.31; 95% Confidence Interval [CI]: 1.07, 1.60). The psychological domain scores were higher in those who used modern contraceptive methods (Coefficient [B] = 0.58; 95% CI: 0.05, 1.11).

**Conclusion:**

This study highlights the importance of the knowledge of modern contraceptive methods to increase their use. Also, the use of modern contraceptive methods improves the psychological QOL domain scores of the migrants using them. These results suggest a need for sexual and reproductive health education for migrants, especially pertaining to modern contraceptive use.

## Introduction

Migration has negative health effects more on women than on men [1] as female migrants face greater disparities in health [2, 3]. In 2015, women comprised about 48% of international migrants out of 244 million international migrants worldwide [4]. Their access to health care is often compromised because of socioeconomic, political, and cultural reasons [5, 6]. They are unable to learn about the available health services in their host countries because of insufficient information before migration, limited language skills, and lack of health insurance [6, 7]. They are also discriminated on the grounds of gender and migrant status [2]. These factors may lead to increased susceptibility to a variety of health problems among female migrants, particularly reproductive illnesses.

Female migrants are at a higher risk of sexually transmitted infections (STIs) and reproductive tract infections (RTIs) than the host country’s female citizens [2, 8]. They are also more likely to experience unintended pregnancies, induced abortions, and poor pregnancy outcomes because of their fewer and later antenatal visits [9–11]. To avoid these risks, modern contraceptives, such as condoms, hormonal methods, and non-hormonal intrauterine devices (IUD), can be useful as they give women a better fertility control [3] and promote gender equality [12]. However, female migrants may not be aware if these methods are available in the host country [8]. Also, women in developing countries are less likely to use them which probably contribute to their lower use even after migration. Thus, these migrants have high fertility rates as most of them do not use modern contraceptives [13].

Among female migrants, they may improve the quality of life (QOL) when they use modern contraceptives [14]. They are likely to have poorer QOL compared to male migrants [15] and female citizens of the host country. The probable reasons are unintended pregnancies and abortions [16]. By using modern contraceptives, they can decrease the fear of unwanted pregnancy compared to using the traditional methods such as withdrawal and rhythm method [17]. They can also improve sexual life and promote psychosocial well-being [18]. In addition, hormonal contraceptives such as oral contraceptive pills (OCP) and progestin-only injectable (Depo-Provera), also provide medical benefits such as regulation of menstrual cycle and blood flow, and decrease in menstrual pain. These factors may help improve the physical health of female migrants and thus, help in improving the QOL of female migrants.

Although modern contraceptive services give benefits to female migrants, they are not covered by the health insurance schemes in many countries including Japan [19, 20]. Thus, many migrants experience difficulty in obtaining these services due to their high costs. On the contrary, family planning is a priority program of Ministry of Health and Population (MoHP) in Nepal [21]. It is a component of the reproductive health care services of the Nepal Health Sector Strategy: 2015–2020 [21]. Family planning services including all modern contraceptives are available free of cost at public health facilities [21], and at subsidized rates at private clinics, hospitals, and drug stores. They include Depo-Provera, IUD, OCPs, implants, male and female sterilization, and male condoms. The family planning program promotes their use since traditional methods are considered less effective than modern methods [17]. However, only 43% married women used modern contraceptives in Nepal in 2016, despite their easy availability. The popular methods used were female sterilization (15%), Depo-Provera (9%), male sterilization (6%), and OCPs (5%) [22].

The Nepalese population is rapidly growing in Japan and was the sixth largest foreign population in 2016 [23]. More than 60,000 Nepalese resided there, of which 21,000 were women [23]. Female migrants may face difficulty finding their preferred choice of contraceptive method as SRH services provided by the Japanese health system may differ from that in Nepal. As a consequence, they are likely to adopt the contraceptive trends in Japan. Married Nepalese female migrants usually come to Japan to accompany their husbands and to earn money [24, 25]. Their access to contraceptive services may be poor due to limited language skills and knowledge of SRH services in Japan. Thus, these migrants are prone to unintended pregnancies and induced abortions which decrease their QOL [16].

Limited evidence is available on the factors associated with the use of contraceptive methods by female migrants in Japan. Moreover, evidence remains scarce on the QOL of female migrants in relation to contraceptive use. As the Nepalese migrant population is increasing in Japan, the SRH issues of these female migrants should be addressed. Thus, the study was conducted to:

1. examine the factors associated with the use of modern contraceptive methods among married Nepalese female migrants in Japan, and
2. explore the association between the use of modern contraceptive methods and QOL among married Nepalese female migrants in Japan.

## Methods

### Study design and settings

A cross-sectional study was conducted among married Nepalese female migrants residing in five prefectures in Japan from August to October, 2016. The prefectures were selected on the basis of higher number of Nepalese migrants residing there in 2016: Tokyo (Nepalese population: 21,005), Kanagawa (4,085), Chiba (4,055), Saitama (3,397), and Tochigi (2,256) [23]. These prefectures accounted for about 57% of the total Nepalese population in Japan.

### Participants

The migrants included in this study were married Nepalese women who were in the reproductive age group of 18–49 years at the time of data collection. They were living with their husbands and had stayed in Japan for more than three months consecutively. However, migrants who were pregnant or intending to have a child within two years, at the time of interview were excluded. Unmarried women were not included in the study due to possibility of biased answers owing to sensitivity of the topic.

### Measures

#### Use of modern contraceptive methods

The migrants were asked about their currently used modern contraceptive methods [26]. It was categorized into two types: modern contraceptives and none. “Modern contraceptives” was coded as “1” and “none” as “0”. “None” included the use of traditional contraceptive methods and non-use of any of the contraceptive methods. Many migrants in this study used more than one method, usually a combination of modern and traditional contraceptive methods. In such a case, only the dominant method of contraception was taken into account.

#### Quality of life

WHOQOL-BREF questionnaire was used to evaluate the QOL of the migrants which consists of 26 items [27]. It is derived from the WHOQOL-100 and has four domains: physical, psychological, social and environmental health. The item scores ranged from 1 to 5, with a higher score indicating better QOL. Nepali language version of the questionnaire is available and has been used previously [28, 29]. The translated Nepali language WHOQOL-BREF questionnaire was tested for validity and showed good internal reliability (Cronbach’s alpha = 0.85) [28]. The Cronbach’s alpha of the overall QOL domain in this study was 0.87 and for different QOL domains were: physical domain (0.73), psychological domain (0.71), social domain (0.75), and environmental domain (0.74).

#### Socio-demographic and migration characteristics

Variables such as age, religion, education, employment, husband’s age, husband’s education, husband’s employment, marital duration, and the number of children were included in the questionnaire. The questions were adopted from the Nepal Demographic and Health Survey 2011 [26]. It also included the following variables: duration of stay in Japan, visa status, husband’s visa status, income in the last month, and Japanese language skills.

The duration of stay in Japan was recorded in completed years and months. However, for data analysis, months were converted to years. Migrants’ visa status was categorized into student, dependent, and others. “Others” included female migrants with visa status such as engineer, specialist in humanities, international services, permanent resident, spouse of permanent resident, researcher, and business manager. Similarly, husbands’ visa status was categorized into skilled labor, student and others. “Others” included husbands with visa status such as dependent, engineer, specialist in humanities, international services, permanent resident, researcher, professor, and business manager. Responses for Japanese language skills were obtained in two categories: “none” and “yes”. “None” included migrants who did not speak Japanese language at all and “yes” included migrants who spoke any level of Japanese language.

#### Knowledge of modern contraceptive methods

The factor “knowledge of modern contraceptive methods” contained 10 items. Each item consisted of questions on various modern contraceptive methods. The responses were recorded as 0 (no) and 1 (yes), and the total score for this factor was calculated by summing all 10 items, scores ranging from 0 to 10. A higher score indicated that the migrants had a higher knowledge of modern contraceptive methods. Cronbach’s alpha for this factor was 0.71. The questions were adopted from the Nepal Demographic and Health Survey 2011 [26].

#### Contraceptive use, family planning, and abortion

For the secondary outcome, the use of modern contraceptive methods was utilized as an exposure variable. The migrants were asked about their methods of contraception. They were also asked about the reason for non-use of modern contraceptives and their history of any induced abortion in Japan. The questions were also adopted from the Nepal Demographic and Health Survey 2011 [26].

### Data collection

A female research assistant was hired for data collection. She was provided with one-day training session to familiarize her with the study objectives, study protocol, research ethics and contents of the questionnaire. Data were collected using interviewer-administered questionnaire via telephone interview between August and October, 2016. Female members of various Nepalese organizations and communities in Japan were contacted to obtain phone numbers of female migrants. Snowball sampling method was employed for this study.

The migrants were contacted via telephone and informed about the study objectives, procedure, and confidentiality. Oral informed consent was obtained and they were contacted again by a telephone call at a scheduled time, for telephone interview. Each interview lasted 20–30 minutes. Out of 250 female migrants approached for telephone interview, 220 voluntarily participated in the study. Data from 8 migrants were excluded due to incomplete questionnaire, and from 23 migrants who were pregnant or intending to have a child within two years at the time of interview. Finally, data from 189 migrants were included for data analyses.

### Questionnaire

A semi-structured questionnaire was prepared in English for data collection based on previous studies [26, 27]. The English questionnaire was translated into Nepali language. Two independent native translators then back-translated the Nepali questionnaire into English. The Nepali questionnaire was finalized after comparing both versions of the questionnaire and after pre-testing it among 10 migrants meeting the eligibility criteria. The questionnaire was refined as per the findings of pretest.

### Data analysis

Simple and multiple logistic regression analyses were conducted to examine the association between the independent variables and the use of modern contraceptive methods. Multiple linear regression analysis was used to examine the association between the use of modern contraceptive methods and QOL. All analyses were performed using Stata version 13. “Husband’s income” was excluded as it indicated multicollinearity with the variable “husband’s age” which had Variance Inflation Factor (VIF) greater than the recommended value of 10.0 [30].

### Ethical considerations

Obtaining written informed consent was difficult in this study because it involved telephone interview as a method for data collection owing to the hard-to-reach nature of migrants [31]. Thus, oral informed consent was obtained to prevent potential breach in migrants’ anonymity and difficulty in reaching them. Their interview was not audio-recorded. However, the migrants were informed about the study objectives, procedures, benefits, risks, and confidentiality using a detailed information sheet and consent protocol before the interview. Participation was voluntary and the migrants could withdraw from this study at any time. The interviewers recorded migrants’ names on an oral consent sheet with their permission. Migrants’ identity was kept anonymous by using identification codes. The study and oral informed consent process were approved by the Research Ethics Committee of the Graduate School of Medicine at The University of Tokyo, Japan. Oral informed consent is also approved by the Ministry of Health, Labour and Welfare, Government of Japan, when the study does not involve any human intervention and invasiveness, and possesses minimal risk of harm [32].

## Results

Table 1 summarizes the socio-demographic and migration characteristics of the Nepalese female migrants living in Japan. The mean age of the migrants was 30.5 years (Standard Deviation [SD] 4.8, range 20.0–43.0). Among 189, 53 (28.0%) were educated below higher secondary level and 52 (27.5%) were educated up to higher secondary level. Remaining 84 (44.5%) had obtained undergraduate degree and above. The mean age of the husbands was 34.6 years (SD 5.1). The mean of marital duration was 8.2 (SD 5.8) years. The number of migrants under dependent visa was 151 (79.8%), followed by student visa at 19 (10.1%), and others at 19 (10.1%).

**Table 1:** Socio-demographic and migration characteristics of migrants (n = 189).

Variables	n	%	Mean (SD)
**Age (in years)**			30.5 (4.8)
**Religion**			
Hindu	165	87.3	
Buddhist	24	12.7	
**Education**			
< Higher secondary	53	28.0	
Higher secondary	52	27.5	
≥ Undergraduate	84	44.5	
**Husband’s age (in years)**			34.6 (5.1)
**Husband’s education**			
< Higher secondary	20	10.6	
Higher secondary	64	33.9	
≥ Undergraduate	105	55.5	
**Marital duration (in years)**			8.2 (5.8)
**Number of children**			
None	68	36.0	
1	49	25.9	
≥2	72	38.1	
**Duration of stay in Japan (in years)**			4.9 (4.2)
**Visa status**			
Student	19	10.1	
Dependent	151	79.8	
Others^1^	19	10.1	
**Employment status**			
None	25	13.2	
Part-time	153	81.0	
Full-time	11	5.8	
**Income per month (in Yen)**			
< 100000	119	63.0	
100000–200000	55	29.0	
> 200000	15	8.0	
**Japanese language skills**			
None	14	7.4	
Yes	175	92.6	
**Husband’s visa status**			
Skilled labor	85	45.0	
Student	17	9.0	
Others^2^	87	46.0	
**Husband’s employment status**			
None	12	6.4	
Part-time	15	7.9	
Full-time	162	85.7	
**Income per month (in Yen)**			
< 100000	19	10.0	
100000–200000	99	52.4	
> 200000	71	37.6	

^1^ It includes female migrants with visa status such as engineer, specialist in humanities, international services, permanent resident, spouse of permanent resident, researcher, and business manager.

^2^ It includes husbands with visa status such as dependent, engineer, specialist in humanities, international services, permanent resident, researcher, professor, and business manager.

Table 2 illustrates the knowledge and use of contraceptive methods among the migrants. All migrants had knowledge of at least one modern contraceptive method. Of 189 migrants, 82 used modern contraceptives dominantly. Among 82, 58 (70.7%) used male condoms and 9 (11.0%) used OCPs. It was followed by IUD use by 7 (8.5%) and implant by 4 (4.9%). The remaining 107 did not use modern contraceptives. Among them, 85 (79.4%) used withdrawal method and only 2 (1.9%) used rhythm method, while 20 (18.7%) did not use any method of contraception.

**Table 2:** Knowledge and current use of contraceptive methods.

**Contraceptive methods (n = 189)**	**Knowledge of contraceptive methods**^1^	
	**n**	**%**
**Modern methods**		
Male condom	189	100.0
OCPs	185	97.9
Depo-Provera	176	93.1
Male sterilization	173	91.5
IUDs	172	91.0
Emergency contraception	152	80.4
Implants	147	77.8
Female sterilization	137	72.5
Female condom	72	38.1
**Traditional methods**		
Rhythm method	186	98.4
Withdrawal	146	77.3
**Others**	2	1.1
**None**	0	0.0
**Contraceptive methods**	**Current use of contraceptive methods**^2^	
	**n**	**%**
**Modern methods (n = 82)**		
Male condom	58	70.7
Oral Contraceptive Pills	9	11.0
Intrauterine Devices	7	8.5
Implants	4	4.9
Male sterilization	3	3.7
Female sterilization	1	1.2
**Traditional methods or none (n = 107)**		
Withdrawal	85	79.4
Rhythm method	2	1.9
None	20	18.7

^1^ Multiple responses obtained.

^2^ Dominant method of contraception asked.

Table 3 shows the main reasons for non-use of modern contraceptives which were: (i) concerns of side effects (46.7%), (ii) high cost (31.8%), and (iii) opposition from husband (24.3%). Moreover, only 10 (5.3%) migrants had visited a health care facility in Japan to obtain information on family planning. During their stay in Japan, 21 (10.4%) had an induced abortion, of which, only 12 (57.1%) visited a health care provider.

**Table 3:** Contraceptive use, family planning, and abortion.

Variables	n	%
**Country of obtaining modern contraceptive methods (n = 82)**		
Nepal	28	34.1
Japan	54	65.9
Reason for not using modern contraceptive methods (n = 107)*		
Concern of side effects	50	46.7
Cost	34	31.8
Husband opposed	26	24.3
Inconvenient to use	18	16.8
Respondent opposed	15	14.0
Infrequent sex	12	11.2
**Visit to a health care center in Japan for family planning (n = 189)**		
Yes	10	5.3
No	179	94.7
**History of abortion in Japan (n = 189)**		
Yes	21	10.4
None	168	89.6
**Procedure used for abortion in Japan (n = 21)**		
Surgical procedure	12	57.1
Medical abortion (Abortion pill)	9	42.9
**Visit to hospital or doctor for abortion in Japan (n = 21)**		
Yes	12	57.1
No	9	42.9

*Multiple responses obtained

Table 4 demonstrates simple and multiple logistic regression analyses of the factors associated with the use of modern contraceptive methods. After adjusting for covariates and confounders, it was seen that migrants who had higher knowledge of modern contraceptive methods were more likely to use them (Adjusted Odds Ratio [AOR] = 1.31; 95% Confidence Interval [CI]: 1.07, 1.60). Similarly, those with longer marital duration were more likely to use modern contraceptives (AOR = 1.16; 95% CI: 1.01, 1.34).

**Table 4:** Simple and multiple logistic regression: Factors associated with the current use of modern contraceptive methods (n = 189).

Variables	Use of modern contraceptive methods	
	OR (95% CI)	AOR^1^ (95% CI)
**Contraceptive knowledge**	1.26 (1.07, 1.48)*	1.31 (1.07, 1.60) *
**Education**		
< Higher secondary	1	1
Higher secondary	1.65 (0.76, 3.58)	1.19 (0.46, 3.09)
≥ Undergraduate degree	2.12 (1.05, 4.29)*	1.10 (0.39, 3.08)
**Husband’s age**	0.94 (0.88, 0.99)*	0.86 (0.74, 1.00)*
**Marital duration**	0.96 (0.91, 1.01)	1.16 (1.01, 1.34)*
**Visa Status**		
Student	1	1
Dependent	0.21 (0.06, 0.76)*	0.34 (0.08, 1.47)
Others	0.70 (0.13, 3.68)	1.45 (0.21, 9.9)
**Employment**		
None	1	1
Employed	0.22 (0.11, 0.87)*	0.80 (0.23, 2.80)
**Japanese language skills**		
None	1	1
Yes	0.22 (0.05, 1.00)*	0.14 (0.02, 0.82)*
**Abortion in Nepal**		
Yes	1	1
No	2.21 (1.05, 4.65)*	1.36 (0.55, 3.38)
**Abortion in Japan**		
Yes	1	1
No	0.39 (0.15, 0.99)*	1.80 (0.38, 2.04)

* p-value < 0.05

^1^ Adjusted Odds Ratio: adjusted for age, number of children, husband’s visa status, husband’s employment status, and visit to health care center in Japan for family planning.

Table 5 shows the QOL domain scores for overall and individual domains. Migrants who used modern contraceptive methods had better QOL scores for all QOL domains besides the environmental domain.

**Table 5:** QOL scores.

QOL domain scores	Mean (SD)	Mean (SD)	Mean (SD)
	Whole QOL	Modern contraceptive methods	Non-modern contraceptive methods
Overall	93.4 (9.1)	94.2 (8.8)	92.7 (9.8)
Physical	14.6 (1.9)	14.7 (1.8)	14.5 (2.0)
Psychological	13.6 (1.9)	14.0 (1.8)	13.3 (2.0)
Social	15.1 (1.9)	15.1 (2.1)	15.0 (1.9)
Environmental	14.7 (1.6)	14.6 (1.5)	14.8 (1.7)

Table 6 illustrates the adjusted factors associated with all the domains of QOL using multiple linear regression models. The psychological domain score was higher for migrants using modern contraceptive methods (Coefficient [B] = 0.58; 95% CI: 0.05, 1.11). The environmental domain score was higher for migrants who had higher knowledge of modern contraceptives (B = 0.17; 95% CI: 0.03, 0.30). Similarly, those without history of abortion in Japan had higher physical (B = 1.93; 95% CI: 0.69, 3.17) and psychological (B = 2.08; 95% CI: 0.90, 3.25) domain scores.

**Table 6:** Multiple linear regression: Association of the use of modern contraceptive methods and other independent variables with WHOQOL domains^a^.

Variables	Physical	Psychological	Social	Environmental
	B (95% CI)	B (95% CI)	B (95% CI)	B (95% CI)
**Modern contraceptive use**	0.19 (-0.36, 0.75)	0.58 (0.05, 1.11)*	0.05 (-0.52, 0.63)	-0.08 (-0.53, 0.38)
**Contraceptive knowledge**	0.10 (-0.07, 0.26)	0.04 (-0.12, 0.20)	0.12 (-0.05, 0.29)	0.17 (0.03, 0.30)*
**Age**	-0.23 (-0.36, -0.11)***	-0.12 (-0.24, 0.01)	-0.22 (-0.35, -0.08)**	-0.05 (-0.15, 0.06)
**Education**				
< Higher secondary^b^				
Higher secondary	0.24 (-0.55, 1.02)	0.74 (0.01–1.49)	0.96 (0.14, 1.78)*	0.95 (0.31, 1.60)**
≥Undergraduate	0.34 (-0.45, 1.13)	0.63 (-0.11, 1.38)	0.95 (0.13, 1.77)*	0.75 (0.10, 1.39)*
**Employment**				
None^b^				
Employed	-0.49 (-1.37, 0.39)	-0.45 (-1.28, 0.39)	-0.96 (-1.88, -0.05)*	-0.90 (-1.63, -0.18)*
**Japanese language skills**				
None^b^				
Yes	1.12 (0.06, 2.18)*	1.67 (0.66, 2.68)**	-0.04 (-1.15, 1.07)	-0.36 (-1.23, 0.51)
**Abortion in Japan**				
Yes^b^				
No	1.93 (0.69, 3.17)*	2.08 (0.90, 3.25)**	0.70 (-0.59, 1.99)	0.07 (-0.95, 1.08)

* p-value < 0.05

** p-value < 0.01

*** p-value < 0.001

^a^ Adjusted for visa status, husband’s age, history of abortion in Nepal, and number of children.

^b^ Reference group

## Discussion

The key findings of this study are: 1) Among the Nepalese migrants in Japan, those who had higher knowledge of modern contraceptive methods were more likely to use them. 2) Those who used modern contraceptives had higher scores for psychological QOL domain whereas those who had undergone induced abortion in Japan had lower psychological domain scores.

This study found that the pattern of contraceptive use by migrants was different from Nepalese women living in Nepal. More women in Nepal used modern contraceptive methods such as female sterilization and Depo-Provera [22, 26]. On the other hand, migrants in Japan mostly used male condoms and withdrawal method which is similar to the pattern of contraceptive use in Japan [33]. Migrants tend to adapt to the contraceptive trends of their host countries [34] which may explain a different pattern of contraceptive use in these migrants.

The utilization of modern contraceptives was better among migrants who had higher knowledge in this study. Similar results were observed in Iran and China [35, 36]. Higher knowledge gives women more options of contraception. Thus, they are more likely to use them. Similarly, migrants with higher knowledge of modern contraceptives had higher environmental domain scores. Women who have a higher level of education have a better social status [37]. Education and employment have shown to increase the environmental QOL domain scores in previous studies [38].

Furthermore, higher psychological QOL domain scores were seen in migrants who used modern contraceptive methods and those with no history of induced abortion in Japan. This could be because their fear of being pregnant may have decreased from better control of their fertility. Migrants are prone to mental health issues as suggested by various studies [39]. Unintended pregnancies and induced abortions may also contribute to poor mental health in female migrants [40, 41].

Similarly, migrants without a history of abortion had higher physical domain scores than those who underwent induced abortions. Migrants, in particular when their status of stay is illegal, are likely to seek harmful methods of induced abortion [42]. Similar behavior is also seen among migrants who had lower literacy rate and poor socioeconomic status. These abortion methods may produce more negative health outcomes [43] compared to legal methods. As seen in the results section, about 10% migrants underwent induced abortion during their stay in Japan among which nine underwent medical abortion without medical supervision. Medical abortion is not a legally approved abortion procedure in Japan. It could be due to inadequate knowledge of availability of contraceptives in Japan and lower socio-economic status [3]. The abortions can also be attributed to the improper use of modern contraceptive methods [44] or the ineffective use of traditional methods [17]. This result highlights the risk of unsafe methods of induced abortion that the female migrants may use to avoid the high costs of legal abortion [45] in the host country.

However, some limitations should be noted in this study. Though confidentiality was assured, information bias could still be possible because of a closed society in Nepal, where women do not discuss openly on the matters related to sexuality and contraception. Thus, migrants could have under-reported or over-reported their use of contraceptives to avoid awkwardness due to further questioning on their use. Also, migrants are hard-to-reach population [31] as it is difficult to find a database to identify them [46]. Moreover, their involvement, particularly female migrants’, in health-related programs is lower compared to the host country’s female citizens [31]. Using self-administered questionnaire for data collection could have captured a larger sample size. However, missing data and poor response rate to questionnaires could pose a problem for the researcher.

Despite these limitations, this study is one of the first studies which identified the factors associated with contraceptive use and QOL in Asian migrants in Japan. Although this study only involved Nepalese female migrants, the results may be applicable to female migrants from other low and middle income countries with similar background such as low literacy rate, poor socio-economic status and low skills.

## Conclusion

In this study, Nepalese female migrants are more likely to use modern contraceptive methods when they have higher knowledge about them. The migrants who used modern contraceptives had better QOL domain scores compared to those who did not use them. These results suggest a need for SRH education programs for migrants, especially pertaining to modern contraceptive use.
